# Gene and lncRNA Profiling of ω3/ω6 Polyunsaturated Fatty Acid-Exposed Human Visceral Adipocytes Uncovers Different Responses in Healthy Lean, Obese and Colorectal Cancer-Affected Individuals

**DOI:** 10.3390/ijms25063357

**Published:** 2024-03-15

**Authors:** Sabrina Tait, Enrica Calura, Antonella Baldassarre, Andrea Masotti, Barbara Varano, Sandra Gessani, Lucia Conti, Manuela Del Cornò

**Affiliations:** 1 Center for Gender-Specific Medicine, Istituto Superiore di Sanità, 00161 Rome, Italy; sabrina.tait@iss.it (S.T.); barbara.varano@iss.it (B.V.); manuela.delcorno@iss.it (M.D.C.); 2 Department of Biology, University of Padua, 35122 Padua, Italy; enrica.calura@gmail.com; 3 Research Laboratories, Bambino Gesù Children’s Hospital-IRCCS, 00146 Rome, Italy; antonellabaldassarre82@gmail.com (A.B.); andrea.masotti@opbg.net (A.M.); 4 CNR Institute of Translational Pharmacology, 67100 L’Aquila, Italy; sandra.gessani@ift.cnr.it

**Keywords:** polyunsaturated fatty acids, adipose tissue, obesity, colorectal cancer, long non-coding RNA, transcriptome

## Abstract

Colorectal cancer (CRC) is a major life-threatening disease, being the third most common cancer and a leading cause of death worldwide. Enhanced adiposity, particularly visceral fat, is a major risk factor for CRC, and obesity-associated alterations in metabolic, inflammatory and immune profiles in visceral adipose tissue (VAT) strongly contribute to promoting or sustaining intestinal carcinogenesis. The role of diet and nutrition in obesity and CRC has been extensively demonstrated, and AT represents the main place where diet-induced signals are integrated. Among the factors introduced with diet and processed or enriched in AT, ω3/ω6 polyunsaturated fatty acids (PUFAs) are endowed with pro- or anti-inflammatory properties and have been shown to exert either promoting or protective roles in CRC. In this study, we investigated the impact of ex vivo exposure to the ω3 and ω6 PUFAs docosahexaenoic and arachidonic acids on VAT adipocyte whole transcription in healthy lean, obese and CRC-affected individuals. High-throughput sequencing of protein-coding and long non-coding RNAs allowed us to identify specific pathways and regulatory circuits controlled by PUFAs and highlighted an impaired responsiveness of obese and CRC-affected individuals as compared to the strong response observed in healthy lean subjects. This further supports the role of healthy diets and balanced ω3/ω6 PUFA intake in the primary prevention of obesity and cancer.

## 1. Introduction

Colorectal cancer (CRC) is the third most common cancer and the second cause of cancer-related mortality worldwide, with its burden expected to increase in the coming years (IARC 2020. Colorectal cancer. Source: Globocan. The Global Cancer Observatory. Available from: http://gco.iarc.fr/today, accessed on 20 January 2024). CRC is a multifactorial disease with both genetic and environment/lifestyle-related etiology. Body weight, dietary habits and physical activity deeply influence cancer risk, with important implications for prevention. Specifically, obesity, resulting from a long-term imbalance between energy intake and expenditure and characterized by increased visceral fat, represents a major predisposing factor for CRC and is also associated with worse disease outcomes [1,2]. The obesity–CRC relationship is complex and multifaceted, and alterations in metabolic, immune, inflammatory and fatty acid (FA) profiles occurring in visceral adipose tissue (VAT) play a crucial role in the generation of obesity-associated inflammation, and contribute to promoting or sustaining intestinal carcinogenesis [3,4,5,6].

The role of nutrition in obesity and CRC has been extensively investigated, and epidemiological and preclinical studies have clearly shown that specific dietary patterns and dietary bioactive compounds can influence overweight, obesity and CRC risk, mainly by regulating inflammation and oxidative stress [7,8]. FAs, introduced with the diet and processed and released by AT, are gaining importance as main players due to their capacity to act in an autocrine and paracrine manner, and to influence both cancer cell proliferation and host immune and inflammatory responses [9,10]. In particular, long-chain ω6 and ω3 polyunsaturated fatty acids (PUFAs) have been associated with pro- and anti-inflammatory pathways, respectively [11], and reported to exert either promoting or protective roles in CRC [7,12]. ω3 PUFAs, increasingly recognized for their health benefits, have become one of the hotspots in nutritional biochemistry research and generated considerable interest as nutritional supplements [13,14]. Indeed, dietary supplementation with ω3 PUFA-rich oils or eicosapentaenoic acid (EPA)/docosahexaenoic acid (DHA) in healthy individuals was associated with the extensive modulation of blood immune cell gene expression, with the regulation of inflammatory and oxidative stress pathways, cell adhesion, the DNA damage response, and glucose and lipid metabolism [10,15,16,17,18,19,20]. The modulation of oxidant/antioxidant balance in PBMC was also reported in a few intervention studies involving obese subjects [10,21,22,23]. Moreover, data on the beneficial effects of ω3 PUFAs on inflammation and the immune response have arisen from studies on in vitro exposed blood immune cells from healthy donors [10]. Contrariwise, treatment with ω6 PUFAs, mainly arachidonic acid (AA), resulted in detrimental effects such as reactive oxygen species and mitochondrial stress generation, de novo lipogenesis and impaired immune responses [10,12]. By virtue of their effects on immunity and inflammation, ω3 and ω6 PUFAs have gained prominence in CRC research as potential modulators of cancer onset and progression, by acting on cancer cells as well as by shaping gut microbiota and immune cell profiles [24]. However, in spite of the crucial role played by AT inflammation in CRC, the impact of PUFAs on AT functions in cancer patients has not been investigated so far. More generally, only a few studies have analyzed the effects of direct PUFA exposure or dietary supplementation on gene expression in human AT (homing both adipocytes and immune cells) in spite of the huge alterations in endogenous FA profiles described in both obesity and CRC [10]. 

Specifically, in intervention studies involving obese individuals, ω3 PUFAs and ω3 PUFA-rich oils or fish were shown to modulate inflammasome and inflammatory cytokine genes, despite the high variability related to the fat depot analyzed (subcutaneous AT (SAT) versus VAT) [25,26,27,28]. Moreover, EPA and DHA supplementation also modulated several inflammation- and immune response-related genes in SAT from healthy individuals following evoked inflammation [29,30]. Conversely, their effects on immune gene expression were less pronounced in obese individuals [31]. Furthermore, the down-regulation of specific inflammatory mediators was described in SAT and VAT explants from obese subjects upon direct stimulation with ω3 PUFAs, with a better response obtained in SAT [32,33,34]. Most of these studies provided information on ω3 PUFAs’ effects on a limited number of genes or gene products in whole AT (mainly SAT) from either diseased subjects or healthy controls. In contrast, comparative studies aimed at deciphering the global transcriptional response to ω3 and ω6 PUFAs, as well as the specific role of adipocytes, are lacking. In this regard, we have previously reported that both ω3 (DHA) and ω6 (AA) PUFAs can modulate the production of cytokines and adipokines and the activation of inflammation-related transcription factors in purified VAT adipocytes from obese and CRC-affected subjects as compared to healthy lean controls [4,5,35].

Accumulating evidence has revealed that gene regulation by non-coding RNAs (ncRNAs), specifically long ncRNAs (lncRNAs), is involved in the occurrence and progression of many major diseases, including obesity and cancer [36], and specific lncRNA profiles have been associated with CRC [37]. High-throughput methods and bioinformatics approaches have significantly contributed to the identification of these new transcripts. However, only a few studies have described lncRNAs in human AT in obesity or CRC [38,39,40,41], and their potential role as targets of PUFA-based interventions has been only poorly explored. 

We recently analyzed RNASeq expression profiles of human visceral adipocytes purified from lean, obese and CRC-affected subjects, and highlighted changes in their transcriptional programs specifically associated with obesity and cancer, or both conditions [35]. Furthermore, specific ncRNA-mRNA networks were identified in these subjects [41].

In this study, we performed a whole-transcriptome analysis aimed at investigating the impact of ω6 and ω3 PUFA (AA and DHA, respectively) treatment on human VAT adipocytes from healthy lean, obese and CRC-affected subjects. High-throughput sequencing of protein coding and lncRNAs, as well as pathway analysis and regulatory network constructions, were employed to identify common and specific PUFA effects. The results highlight impaired responsiveness of obese subjects and CRC patients to PUFA stimulation, particularly to DHA, in the face of a robust response of healthy individuals.

## 2. Results

### 2.1. Arachidonic and Docosahexaenoic Acids Differently Affect Adipocyte Transcription in Healthy Lean, Obese and CRC-Affected Subjects

We previously reported that obese and CRC-affected subjects exhibit alterations in VAT PUFA composition and adipocyte gene expression with respect to healthy lean individuals [4,5,35,41]. Moreover, VAT adipocyte exposure to AA or DHA was found to differently modulate the expression and activation of specific factors related to AT inflammation [4,5,35].

To more deeply investigate the impact of pro- and anti-inflammatory PUFAs on VAT adipocyte whole transcription, adipocytes isolated from a subset of previously analyzed lean, obese and CRC subjects [35,41] were left untreated or exposed to AA or DHA, and then, subjected to RNASeq and differential transcript analysis.

The number of differentially expressed (up- and down-modulated) transcripts, including protein-coding transcripts and lncRNAs, following PUFA treatments are summarized in Table 1.

As shown in Table 1, adipocytes from healthy lean (normal weight, NW), obese (OB) and CRC-affected (CRC) individuals exhibit a different transcriptional response to PUFA stimulation. In particular, the highest overall responsiveness to both AA (468 transcripts) and DHA (456 transcripts) was observed in adipocytes from healthy NW subjects, with most of them being up-regulated. Conversely, a lower number of modulated transcripts was found following the AA treatment of cells from OB (352 transcripts) and CRC (270 transcripts) subjects. Notably, in the latter groups, the transcriptional response to DHA was markedly impaired, with approximately 70% (OB) and 60% (CRC) fewer transcripts detected compared to the control NW group (Table 1). Most of the differentially expressed transcripts are represented by protein-coding RNAs, sometimes with two transcripts for the same gene; however, several lncRNAs are also modulated by PUFAs (Table 1) and are discussed in detail below. 

To validate the RNASeq data, we analyzed a panel of six genes randomly picked from NW DEGs by real-time quantitative PCR (qPCR) (Supplementary Data S1). We focused on the NW condition as it was the most affected by PUFAs. Real-time qPCR analysis overall confirmed the RNASeq data in NW subjects, with a borderline significant positive correlation (R^2^ = 0.7726, *p*-value = 0.0717) only in the NW-AA group. In addition, in the OB and CRC groups, some modulations, not detected in the RNASeq analysis, were found to be significant by qPCR, according to the higher sensitivity of this independent technology. 

**Figure 1:**
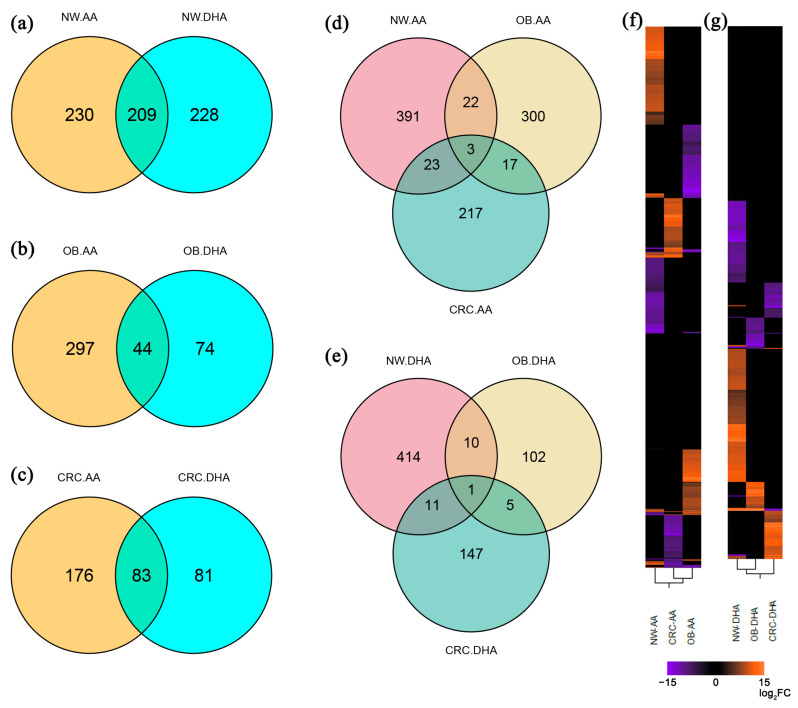
Analysis of transcripts shared by PUFA treatments and by health status or that are unique for each condition. (**a**–**e**) Venn diagrams illustrating overlaps in differentially expressed genes (DEGs) after exposure to AA or DHA of adipocytes from (**a**) lean (NW), (**b**) obese (OB) and (**c**) CRC-affected (CRC) subjects or the three subject categories exposed to AA (**d**) or DHA (**e**). (**f**,**g**) Hierarchical clustering of transcripts in NW, CRC and OB adipocytes treated with AA (**f**) or DHA (**g**). Shades of purple and orange indicate the level of down- and up-regulation, respectively.

Venn diagrams comparing AA and DHA treatments in the same subject group (Figure 1) evidenced an equal distribution of unique and shared DEGs in the NW group, whereas the majority of DEGs were uniquely modulated by AA or DHA in the OB and CRC categories. According to what is described above, the number of transcripts uniquely modulated by DHA in OB and CRC subjects was 60–70% smaller than in the NW group (Figure 1a–c). The discrepancies in the number of transcripts between Table 1 and Figure 1 are due to multiple transcripts associated with the same genes, which are joined in the Venn diagram. Upon comparing all subject groups exposed to the same PUFAs, very limited numbers of overlapping DEGs were found (Figure 1d,e), pointing to a different impact of PUFAs on adipocyte transcription depending on the patient’s health status. Accordingly, the hierarchical clustering of transcripts highlighted how distant the adipocyte transcriptomic profiles are in the different subject categories even following exposure to the same PUFAs (Figure 1f,g).

### 2.2. Pathway Enrichment Analysis of Differentially Expressed Genes Reveals Higher Number of PUFA-Modulated Pathways in Healthy Lean Individuals Compared to Obese and CRC-Affected Subjects

PUFAs can modulate many signal transduction mechanisms of critical importance in adipocyte biology, such as antioxidant, metabolic and inflammatory processes [42]. Therefore, the identified DEGs between the PUFA-treated and untreated groups were explored for their enrichment in Reactome pathways. 

**Figure 2:**
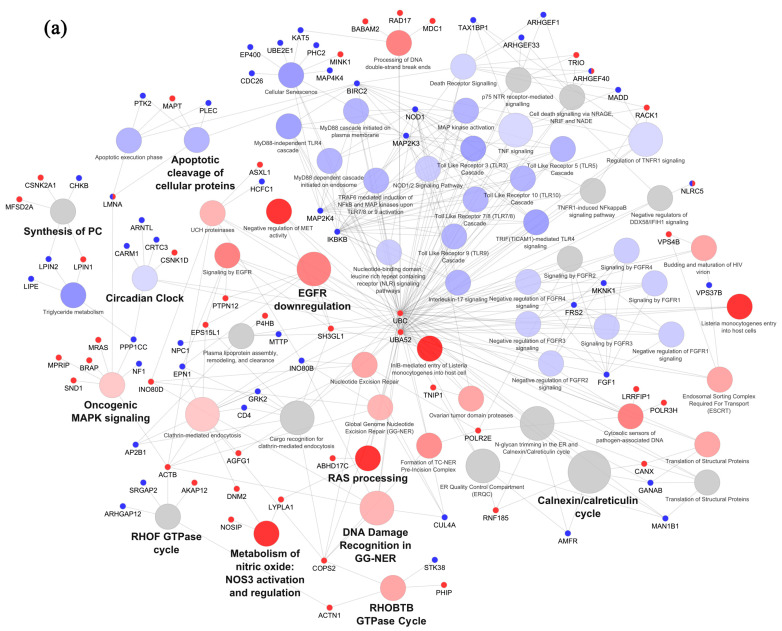

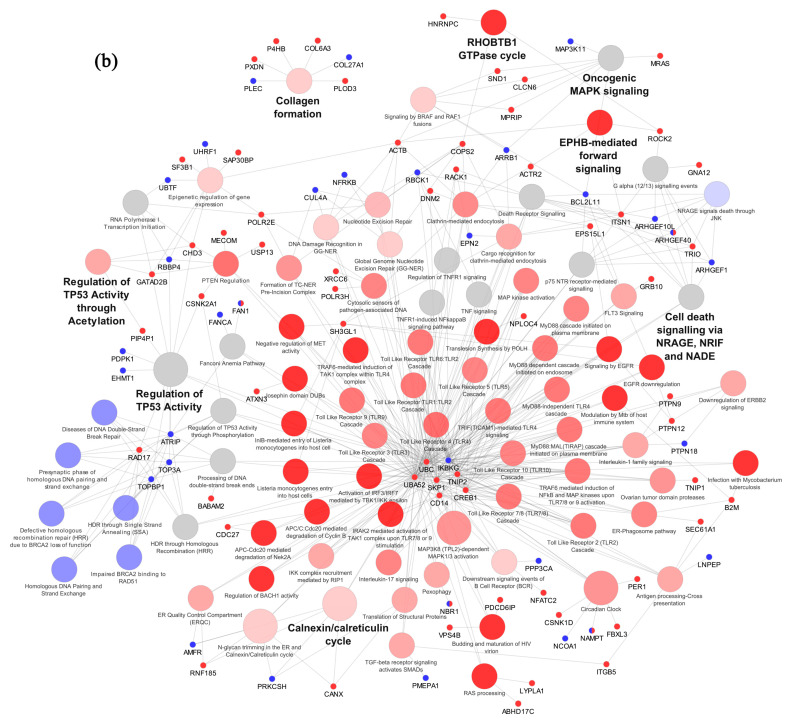

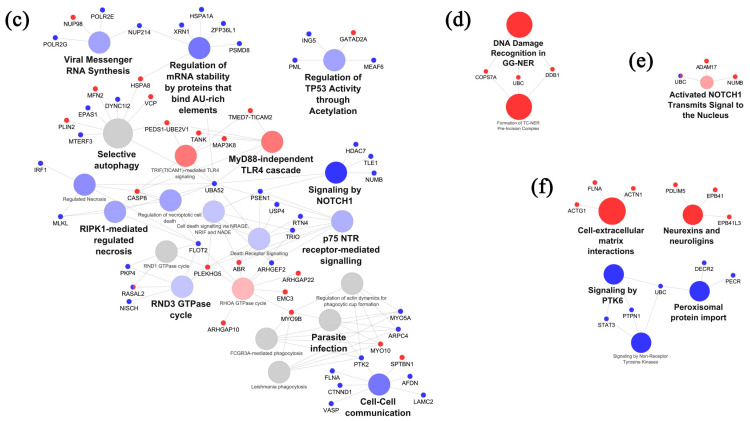
Pathway analysis of PUFA-modulated significant processes. Reactome pathways enriched in adipocytes of healthy lean (**a**,**b**), obese (**c**,**d**) and CRC-affected individuals (**e**,**f**) following treatment with AA (**a**,**c**,**e**) or DHA (**b**,**d**,**f**). Sizes of the circles identifying the pathways are proportional to their statistical significance; shades of red or blue indicate that >50% of DEGs featured in the pathway are up-regulated or down-regulated, respectively. Grey circles indicate that the same numbers of up- and down-regulated DEGs are included in the pathway. DEGs are red or blue according to their up- or down-regulation.

According to the number of DEGs, PUFA treatments affected a huge number of pathways in healthy lean individuals compared to the other subject groups (Figure 2).

In particular, 63 and 85 pathways were significantly enriched in adipocytes from NW individuals by AA and DHA treatment, respectively (Figure 2a,b). AA treatment showed a prevalent repressing effect on the regulated pathways, whereas DHA behaved mostly as an inducer. Deregulated pathways were mainly associated with inflammatory signaling, AT physiology and metabolism or involved in cancer. Among these, the *Calnexin/calreticulin cycle*, associated with a wide variety of signaling processes, such as adipocyte differentiation, cellular stress and the immune response [43], was the most significantly affected pathway by both PUFAs, with a slight prevalence of up-modulation following DHA exposure. Also, the *Circadian clock* pathway was modulated in NW subjects by both ω3 and ω6 PUFAs in opposite directions, with a prevalence of genes repressed or activated by AA or DHA, respectively. The regulation of circadian rhythm genes may play an important role in AT function and homeostasis as well as in tumor initiation. Interestingly, among such genes, PER1 expression was specifically enhanced by DHA treatment, whereas ARNTL and CARM1 were repressed by AA. Notably, all the pathways involving *Toll-like receptor signaling*, related to the immune response to commensal or pathogenic microorganisms, were differently modulated by AA and DHA, further supporting the opposite roles of ω3 and ω6 PUFAs in immunity.

Concerning pathways selectively regulated by ω3 or ω6 PUFAs in NW subjects, DHA specifically regulated terms associated with immunity and infection (e.g., *antigen processing*, *IL-1 signaling*), cancer-related pathways (e.g., *TP53 activity*, *PTEN regulation*, *TGFβ signaling*), as well as extracellular matrix organization and fibrosis (e.g., *collagen formation*). Conversely, categories related to metabolism (e.g., *Triglyceride metabolism*, *the synthesis of PC*), inflammation and oxidative stress (e.g., *TNF and Interleukin-17 signaling*, *the metabolism of nitric oxide*), and signaling pathways that play pivotal roles in the oncogenic process (e.g., *FGFR1-4 signaling*, *NOD1/2 signaling*) were specifically modulated by AA. AA treatment was also found to promote the *oncogenic MAPK pathway* and EGFR signaling (e.g., *signaling by EGFR*, *EGFR down-regulation*), previously involved in AT dysfunction.

In OB and CRC conditions, very few pathways were modulated following adipocyte exposure to both PUFAs (Figure 2c–f). In obese subjects, 21 and 2 pathways were affected by AA and DHA, respectively (Figure 2c,d). Similar to what was observed in NW subjects, AA had a general gene-repressing effect and mostly deranged pathways related to energy balance (e.g., *p75 NTR receptor-mediated signaling*, *RND1* and the *3 GTPase cycle*), metabolism (e.g., *signaling by NOTCH*) and necrosis/apoptosis (e.g., *cell death signaling* via *NRAGE*, *NRIF and NADE*, *death receptor signaling*, *regulated necrosis*). On the other hand, genes related to the *RhoA GTPase cycle* and *TLR4 signaling*, known for their role in the inflammatory response in human adipocytes, were up-regulated by AA in these subjects. Furthermore, the most significant pathway was *selective autophagy* featuring a balanced number of up- and down-regulated genes, linked to other pathways involved in necrosis and cell death signaling, some of which were also similarly repressed by AA in NW adipocytes. As shown in Figure 2d, DHA treatment only promoted two pathways related to DNA damage repair, potentially linked to carcinogenesis, in the OB group. The impact of both PUFAs on adipocyte processes was found to be even less pronounced when CRC patients were analyzed (Figure 2e,f). Indeed, in this group, AA promoted only and specifically the *NOTCH signaling* pathway, as also found in NW-AA group, while DHA modulated a couple of processes, including the *cell–extracellular matrix interactions*, *peroxisomal protein import* and the *signaling by non-receptor tyrosine kinase* pathways. In the latter pathway, STAT3 gene is a key player involved in the adipocyte transcriptional program and in pro-inflammatory pathways, and we previously reported that AA and DHA differently modulate its activation in adipocytes from CRC patients [4]. 

A detailed list of the Reactome pathways enriched by AA and DHA in the different subject categories, along with the genes involved in each term, is reported in Supplementary Data S2.

### 2.3. LncRNA Profiles and mRNA-lncRNA-RNA-Binding Protein Networks Are Specifically Associated with PUFA Stimulation in Healthy Lean, Obese and CRC-Affected Subjects

As lncRNAs have been found to play important roles in the regulation of cancer and AT functions, we also analyzed lncRNA profiles and their potential interaction with DEGs in each condition. Although the implication of regulatory RNAs in human adipocytes remains largely unknown, we have previously described several lncRNAs aberrantly expressed in visceral adipocytes from obese and CRC subjects [41]. LncRNAs that were significantly up- and down-regulated in the three categories of subjects in response to ω3 and ω6 PUFAs are shown in Table 2. Similar to what was observed for protein-coding transcripts, an equal number of lncRNAs was found to be deregulated by AA and DHA in NW subjects. Likewise, the OB and CRC categories were less responsive to DHA, with only two lncRNAs modulated in each group (Table 1 and Table 2). Among the identified differentially expressed lncRNAs (DELs), six were novel transcripts with still unknown functions.

In NW individuals, among the 23 DELs modulated by PUFAs, 5 DELs (SNGH11, SNHG17, LINC00174, LINC01106, TRIM52-AS1) were common to both treatments and modulated in the same directions, 7 were exclusively expressed upon AA treatment and 6 in response to DHA. In OB subjects, 12 DELs were specifically altered in the AA group, while only LIPE-AS1 was specific to DHA. Likewise, in the CRC category, the majority of lncRNAs were selectively modulated following AA exposure. Overall, PUFA type- and health condition-specific lncRNA profiles were found.

**Figure 3:**
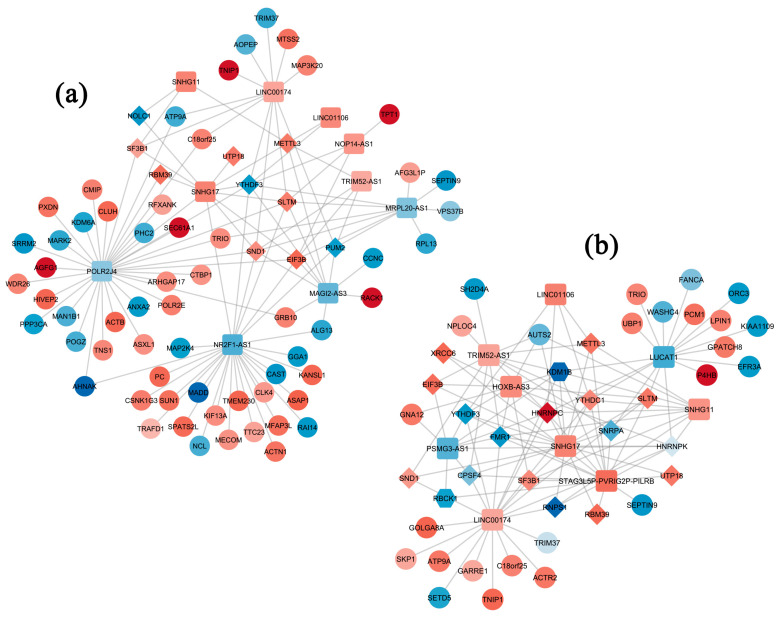

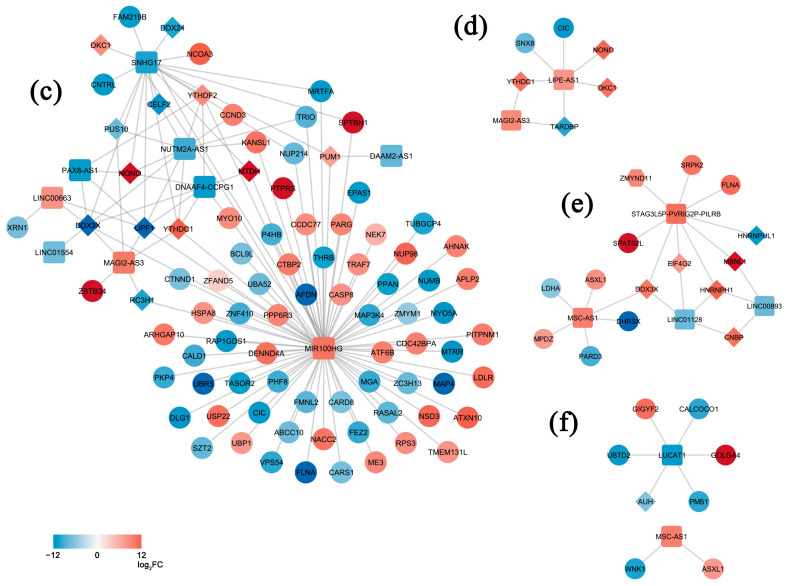
Interactome analysis in response to PUFA exposure. Interaction networks between DELs, DEGs and RBPs in adipocytes of (**a**,**b**) normal weight (NW), (**c**,**d**) obese (OB) and (**e**,**f**) CRC-affected (CRC) subjects treated with AA (**a**,**c**,**e**) or DHA (**b**,**d**,**f**). LncRNAs are represented by rounded rectangles, mRNAs by circles and RBPs by diamonds. Shades of red and blue indicate the level of up- and down-regulation, respectively.

Next, to obtain further insight into the mechanisms underlying the specific effects of PUFAs, validated interactions among differentially expressed lncRNAs, mRNAs and RNA-binding proteins (RBPs) were retrieved from the ENCORI and RNAInter repositories, and the corresponding networks were designed within Cytoscape (Figure 3).

The networks obtained for NW individuals were highly populated, with 80 and 52 nodes for AA and DHA treatment, respectively. The top six hubs in NW-AA were DELs with a degree > 10, followed by four relevant RBPs with a degree > 7, whereas in the NW-DHA network, the top eight interacting ones were DELs with a degree > 9 followed by six RBPs with a degree > 5. In the OB-AA interactome, featuring 95 nodes, MIR100HG appeared to be a highly relevant hub, establishing 77 interactions. Also, SNHG17 and NUTMA2A-AS1 had a degree > 10. The OB-DHA network featured only eight nodes and had only the DEL LIPE-AS1 with a degree > 5. Finally, the interactome for the CRC-AA condition included 19 nodes, with only two DELs having a degree > 5, whereas 10 nodes were present in the CRC-DHA network, with a prevalent role of LUCAT1. 

## 3. Discussion

Obesity and CRC, whose prevalence is constantly increasing, have become very concerning public health issues. These multifactorial disorders are strongly interconnected, and their relationship is also reinforced by their shared AT inflammation and the crucial role played by nutrition [3,44]. AT’s association with cancer is based not only on epidemiological evidence but also on the fact that adipocytes are a main component of the tumor microenvironment in certain cancers, such as malignant breast and gastrointestinal tumors [45,46,47]. Specifically, VAT plays an important role in the establishment of obesity-associated cancer due to its privileged localization to portal circulation and its capacity to secrete key bioactive molecules [48]. Moreover, VAT represents the main site for the processing and release of FAs, which have the potential to control host surveillance mechanisms and shape anticancer responses [10]. 

In this study, we report the results of transcriptomic analysis performed on VAT adipocytes, isolated from healthy lean, obese and CRC-affected individuals exposed ex vivo to ω3 or ω6 PUFAs. Although the effects of PUFAs on AT have been investigated in some studies, to our knowledge, this is the first study comparing VAT adipocytes’ global transcriptional response to AA or DHA treatment in healthy lean individuals versus obese subjects and CRC patients. The results achieved indicate that DHA and AA induce different transcriptional changes depending on the subject category, and define multiple pathways and processes that are specifically altered in response to ω3 or ω6 PUFAs or common to both treatments. 

Previous studies, mainly analyzing whole AT from either obese or lean subjects, have reported the ω3 PUFA-induced modulation of inflammatory genes or gene products, highlighting a more vigorous response in SAT as compared to VAT [5,25,28,29,30,32,33,34]. However, some studies failed to detect any relevant gene modulation in AT from obese subjects following direct exposure or dietary supplementation with ω3 PUFAs [26,27]. Apart from the opposite effects reported for EPA and AA on in vitro adipocyte differentiation and mitochondrial functions [49], very little is known on the AT or adipocyte response to ω6 PUFAs.

We compared ω3 and ω6 PUFA-exposed VAT adipocytes from obese and healthy lean individuals, and extended the analysis of PUFAs’ effects to CRC-affected subjects. In line with previous findings, we definitively show that the most extensive and relevant changes induced by both PUFA types occur in healthy controls, involving pathways and processes which regulate AT homeostasis, metabolism and inflammation, as well as pathways related to cancer. Conversely, adipocytes from obese individuals, and even more from CRC patients, show only a weak transcriptional response to both PUFAs, particularly to DHA, with a much smaller number of genes and pathways modulated. Furthermore, while DHA pathways mainly feature up-regulated genes, AA pathways mainly include down-regulated genes.

Interestingly, in healthy individuals, both PUFAs differentially regulate *circadian clock* and *calnexin/calreticulin cycle*, among the most significant and representative pathways. In this regard, the local circadian clocks present in adipocytes modulate many essential AT processes, including lipolysis, adipogenesis, inflammation, as well as the expression and secretion of adipokines [50], whereas their dysregulation has been identified as a major contributor to carcinogenesis and tumor growth [51]. Furthermore, our results provide evidence of a connection between specific nutrients and the circadian clock in AT, as recently reported [52]. At the same time, the pathway *calnexin/calreticulin cycle* plays a crucial role in adipocyte differentiation, cellular stress and immunity [43]. Indeed, Boden and colleagues found that ER stress-related unfolded proteins, including calnexin and calreticulin, are up-regulated in the SAT of obese subjects [53]. 

We also demonstrate that, although to a lower extent compared to lean individuals, AA treatment is able to affect adipocyte transcription in obese subjects, with particular effects on selective pathways regulating energy balance, metabolism and inflammation. In this regard, it has been reported that AA supplementation can exacerbate high-fat-diet-induced obesity and accelerate its downstream effects in animal models [54]. Conversely, DHA exposure only promotes two statistically significant pathways in adipocytes from obese individuals, in line with the impaired response to ω3 PUFA supplementation recently described in SAT [31]. Similarly, the response to DHA is markedly reduced in CRC patients, suggesting that obesity and CRC, both characterized by chronic AT inflammation, possibly share impaired responsiveness to anti-inflammatory FA.

We recently identified a number of ncRNAs aberrantly expressed in visceral adipocytes from obese and CRC subjects as compared to healthy lean controls, reporting the first analysis of annotated and novel lncRNAs, not previously described in human AT [41]. As further evidence, in this study, we highlight the capacity of AA and DHA to selectively modulate the expression of several lncRNAs, most of which have a recognized role in cancer, depending on the PUFA type and subject group. 

Of note, in both NW and CRC-affected subjects, DHA down-regulates LUCAT1, known for its oncogenic activity [55] and previously found to be up-regulated in obesity [41]. In NW subjects, DHA also down-regulates PSMG3-AS1, reported to be highly expressed in gastric cancer patients [56]. Likewise, AA modulates lncRNAs previously described to have an emerging role in intestinal tumorigenesis, such as NR2F1-AS1 [57], POLR2J4 [58], NOP14 [59] and MAGI2-AS3 [60]. Similarly, AA regulates the expression of SNHG17 and MIR100HG, known to be deregulated in various human cancers [61,62,63,64] and in obesity, while DHA specifically up-regulates LIPE-AS1, a regulator of adipogenesis in animal models [65]. Importantly, in CRC-affected patients, AA specifically down-regulates LINC00893, known to act as a tumor suppressor in CRC [66]. 

Overall, our results show that, similar to what was observed for gene expression, DHA and AA have a more pronounced capacity to modulate lncRNAs in adipocytes from healthy controls as compared to those from obese and CRC subjects. The expression of lncRNAs with cancer-regulating properties in VAT adipocytes, and their modulation by PUFAs, may suggest that they have a relevant role in CRC development and/or progression in light of their potential to be released and to affect distal tissues [67]. Moreover, our network analysis identifies novel potential gene–lncRNA–RBP interactions, with most PUFA-modulated lncRNAs acting as network hubs. Although some scattered data are emerging on the cross-talk between PUFAs and regulatory RNAs in other systems, our findings highlight novel protein-coding lncRNA regulatory circuits controlled by PUFAs in AT, providing further evidence of the central role of this tissue in linking diet to obesity and cancer. 

The results obtained in this study through an omics approach and computational analysis contribute to the identification of candidate genes, ncRNAs and their regulatory networks relevant to many AT biological processes in response to PUFAs, although the direct causality remains to be established. Of note, the focus on adipocytes, the most transcriptionally active and functional cell type in AT [68], represents added value, albeit with difficulties due to the requirement for fresh tissue and the low number of biological replicates. Despite these limitations, our findings highlight new potential players in diet–AT cross-talk, and pave the way for future experimental and functional studies, potentially including larger subject groups and sex-stratified analyses aimed at more deeply characterizing the mechanisms of the adipocyte response to PUFAs. In this regard, relevant focus should be placed on lncRNAs, as emerging evidence links them to numerous obesity-related disorders and multiple types of cancer [39,41,69,70]. 

We and other groups previously reported a reduced ω3/ω6 PUFA ratio in VAT, with the accumulation of AA as well as of saturated FA, as a common feature of obese and CRC-affected individuals [4,5,71,72,73,74]. Moreover, circulating free FAs were found to be increased in these subject categories [75,76,77]. Based on this evidence, we might speculate that the autocrine adipocyte stimulation by endogenous pro-inflammatory FA, as well as by other inflammatory stimuli, occurring in obesity and CRC could result in impaired responsiveness to exogenous stimulation with PUFAs, particularly those endowed with beneficial anti-inflammatory potential. In support of this hypothesis, it has been recently reported, in both mice and humans, that the high production of endogenous FA, also known as de novo lipogenesis, occurring in metabolic diseases and widely demonstrated in both obesity and CRC [78], can negatively affect exogenous ω3 PUFA uptake, thus reducing the outcome of PUFA supplementation [79]. Accordingly, ω3 PUFA intervention was found to decrease pro-inflammatory oxylipins and immune gene expression in SAT to a greater extent in lean as compared to obese individuals [31]. 

ω3 PUFA hypo-responsiveness might represent a limitation for their use as adjuvants in the treatment of metabolic diseases or cancer. Conversely, our results and data from the literature indicate that a robust response can be triggered in healthy individuals, according to the protective effects on CRC and obesity risk reported for these PUFAs in epidemiological studies [12,80,81]. This strongly supports the relevance of healthy diets and the importance of consuming the recommended ω3/ω6 PUFA ratio in the primary prevention of obesity and associated diseases. The nutritional prevention of metabolic diseases continues to hold much promise, and the high CRC preventability highlights the importance of modifiable lifestyle factors, including diet. In this scenario, the regulatory action of dietary PUFAs on AT genomics provides further evidence of a role of diet in the modulation of AT biology, and increases our mechanistic understanding of the role of individual PUFAs, potentially enabling the translation of nutritional interventions into effective risk reduction measures.

## 4. Materials and Methods

### 4.1. Ethics Statement

This investigation was conducted in accordance with ethical standards and with the Declaration of Helsinki, and according to national and international guidelines. It was approved by the Institutional Review Board of Istituto Superiore di Sanità (protocol PRE 838/13-CE 13/410 on 19 December 2013). All enrolled subjects were provided with complete information about the study and asked to sign an informed consent form. 

### 4.2. Patient and Sample Collection

Human VAT was collected from age-matched lean and obese subjects undergoing abdominal surgery or laparoscopy for benign conditions (i.e., gallbladder disease without icterus, umbilical hernia and uterine fibromatosis) and from newly diagnosed CRC patients (histologically proven primary colon adenocarcinoma, stage TNM I-II). The exclusion criteria were as follows: clinical evidence of active infection, recent (within 14 days) use of antibiotics and anti-inflammatory drugs, pregnancy, hormonal therapies, severe mental illness, autoimmune diseases, family history of cancer, other neoplastic diseases. The mean BMI was 23 Kg/m^2^ for the normal weight group, 35 Kg/m^2^ for the obese group and 23.3 Kg/m^2^ for the CRC group. Three subjects/category were analyzed. The anthropometric characteristics of subjects included in the study are reported in Supplementary Data S3.

### 4.3. Adipocyte Isolation/Culture and RNA Preparation

Adipocytes were isolated from VAT as previously described [35]. For each group of individuals (*n* = 3), isolated cells were cultured in low-glucose Dulbecco’s modified Eagle’s medium and left untreated or stimulated for 18 h at 37 °C with docosahexaenoic acid (DHA, 10 μM; Sigma Aldrich, St. Louis, MO, USA) or arachidonic acid (AA, 5 μM; Cayman Chemical Company, Ann Arbor, MI, USA) as previously described [6]. DHA and AA were dissolved under a nitrogen atmosphere in 100% ethanol to make 10 mM stock solutions, which were stored at −20 °C. Stock solutions were diluted in culture media prior to cell treatment. Final concentration of ethanol in treated cells was less than 0.1%. Total RNA was isolated with a Total RNA Purification Plus Kit (Norgen Biotek, Thorold, ON, Canada). RNA quality and quantity were assessed by an Agilent 2100 Bioanalyzer and samples stored at −80 °C until use. Total RNA (2 μg) was used to prepare the library for Illumina sequencing. Single-end reads (>10 M reads per sample) were produced by Illumina HiSeq 2000 as previously described [35,41]. 

### 4.4. RNASeq Data Preprocessing and Differential Expression Analysis

Transcriptome reconstruction and differential expression analysis were performed using the Tuxedo protocol as previously described [35]. Reads were mapped to the human genome (hg38.p2 version) using HISAT2 [82]. Human genome and annotations of reference genes and transcripts (Ensembl 79) were provided as input data. Alignments were then elaborated by StringTie [83], which assembled and quantified the transcripts in each sample. Subsequently, the sample-specific assembled transcriptomes were merged together by a dedicated StringTie module, which created a uniform set of transcripts for all samples. The Gffcompare program was used to compare the genes and transcripts to the existing annotations [84]. We used RSEM to estimate transcript abundances. Transcripts with <10 counts in at least 60% of samples per group of subjects were filtered out. Differential expression (pairwise comparisons) was computed in R v3.9 [85] using edgeR v3.18.1 [86], and a batch effect correction was applied using RuvSeq v1.18.0 [87], with factors of unwanted variation optimized to k = 7. Multiple-testing controlling of the False Discovery Rate (FDR) was performed applying the Benjamini–Hochberg method. Transcripts with a corrected *p*-value (FDR) ≤ 0.05 and log2(Fold Change) ≥ |2| were considered differentially expressed. 

Re-annotation of previously unknown transcripts was performed using BioMart v2.46.3 [88] in R to identify a higher number of lncRNAs. For lncRNAs that were still not annotated, the lncBook nomenclature was used (https://ngdc.cncb.ac.cn/lncbook/, last access 28 November 2023). Venn diagrams were generated with ggvenn v0.1.10 in R, whereas hierarchical clustering was performed with JMP 10.0 (SAS Institute Inc., Cary, NC, USA).

Raw sequence data will be made available on the NCBI Sequence Read Archive (SRA) (http://www.ncbi.nlm.nih.gov/sra, accessed on 3 March 2024); the accession number is PRJNA1082685.

### 4.5. mRNA-lncRNA Regulation Network Construction

The ENCORI [89] the RNAinter [90] databases were used to search for verified DEL-RNA (including RNA-binding proteins) interactions. The overall targets were filtered against the lists of DEGs and integrated to design specific interaction networks for each condition with Cytoscape v3.9.1 software [91]. 

### 4.6. Functional Analysis

The list of DEGs for each condition was examined for significantly enriched pathways by the Cytoscape plug-in ClueGO v2.5.7 [92], querying the Reactome database and applying the Benjamini–Hochberg correction with *p* < 0.05. 

### 4.7. Real-Time Quantitative PCR Validation of Differentially Expressed Transcripts

Gene expression profiling was performed using a custom-made Taqman^®^ low-density array (TLDA), as previously described [35]. Briefly, the synthesis of cDNA from 1 μg of total RNA was performed using a High-Capacity cDNA kit (Applied Biosystems, Waltham, MA, USA) following the manufacturer’s instructions. cDNA was mixed with 2 × TaqMan Universal PCR Master Mix (Applied Biosystems), loaded on a TLDA card and run on a QuantStudio 12 K Flex Real-Time PCR System (Applied Biosystems) following the manufacturer’s instructions. Gene expression values were normalized to the expression of GUSB (selected house-keeping gene). For each sample, the relative gene expression level was determined according to the 2^−ΔΔ*CT*^ method. The primer sequences are listed in Supplementary Data S4. Statistical differences between PUFA-treated samples and basal values were calculated by one-way analysis of variance (ANOVA) with LSD post hoc tests by using SPSS software (Ver.20). Concordance between qPCR and RNASeq data was determined by Pearson’s correlation coefficient analysis in JMP.

## Figures and Tables

**Table 1:** Effect of AA and DHA treatment on adipocyte transcript expression. The numbers of up- and down-differentially (treated vs. untreated) expressed total transcripts, protein-coding genes (differentially expressed genes, DEGs) and lncRNAs (differentially expressed lncRNAs, DELs) in adipocytes exposed ex vivo to AA or DHA are reported (*n* = 3 subjects/category, FDR < 0.05). NW, healthy lean; OB, obese; CRC, CRC-affected subjects.

		Total Transcripts			DEGs			DELs		
Subject category	Treatment	Up	Down	TOT	Up	Down	TOT	Up	Down	TOT
NW	AA	272	196	468	201	150	351	7	5	12
	DHA	280	176	456	265	167	432	7	4	11
OB	AA	159	193	352	152	184	335	6	7	13
	DHA	57	62	119	54	61	115	2	0	2
CRC	AA	144	126	270	138	114	252	2	5	7
	DHA	95	73	168	92	69	161	1	1	2

**Table 2:** LncRNA profiles in response to PUFAs. Differentially expressed lncRNAs (DEL) in normal weight (NW), obese (OB) and CRC-affected (CRC) individuals treated with DHA or AA compared to untreated control. Values are log_2_FC (FDR < 5%).

	NW		OB		CRC	
	AA	DHA	AA	DHA	AA	DHA
Annotated lncRNAs						
DAAM2-AS1			−7.56			
DNAAF4-CCPG1			−10.76			
FAM88B					−8.61	
HOXB-as3		8.34				
LINC00174	6.40	6.31				
LINC00663			7.84			
LINC00884			8.65			
LINC00893					−7.22	
LINC01106	8.54	9.19				
LINC01128					−7.30	
LINC01554			−7.02			
LIPE-AS1				7.61		
LUCAT1		−8.84				−10.57
MAGI2-AS3	−7.67		9.66	8.41		
MIR100HG			10.06			
MRPL20-AS1	−6.14					
MSC-AS1					9.36	9.24
NOP14-AS1	7.51					
NR2F1-AS1	−8.53					
NUTM2A-AS1			−8.84			
PAX8-AS1			−10.64			
POLR2J4	−5.80					
PSMG3-AS1		−8.17				
SNHG11	8.91	7.75				
SNHG17	9.02	8.96	−8.26			
STAG3L5P-PVRIG2P-PILRB		10.54			−8.06; 10.46	
TICAM2-AS1	6.53					
TRIM52-AS1	6.01	6.51				
Novel lncRNAs						
HSALNG0010842			−6.76			
HSALNG0056067		−8.63				
HSALNG0088518			6.11			
HSALNG0101929			6.68			
HSALNG0114326		−11.64				
HSALNG0132632	−6.07					

## Data Availability

SRA accession number: PRJNA1082685.

## Supplementary Materials

The following supporting information can be downloaded at: https://www.mdpi.com/article/10.3390/ijms25063357/s1.

## References

[1] Keum N., Giovannucci E. (2019). Global burden of colorectal cancer: Emerging trends, risk factors and prevention strategies. Nat. Rev. Gastroenterol. Hepatol..

[2] Kumavath R., Pavithran H., Paul S., Anju V.T., Busi S., Dyavaiah M. (2024). Effects of gut microbiome and obesity on the development, progression and prevention of cancer (Review). Int. J. Oncol..

[3] Mitchelson K.A.J., O’Connell F., O’Sullivan J., Roche H.M. (2024). Obesity, Dietary Fats, and Gastrointestinal Cancer Risk-Potential Mechanisms Relating to Lipid Metabolism and Inflammation. Metabolites.

[4] D’Archivio M., Scazzocchio B., Giammarioli S., Fiani M.L., Varì R., Santangelo C., Veneziani A., Iacovelli A., Giovannini C., Gessani S. (2013). ω3-PUFAs exert anti-inflammatory activity in visceral adipocytes from colorectal cancer patients. PLoS ONE.

[5] Del Cornò M., D’Archivio M., Conti L., Scazzocchio B., Varì R., Donninelli G., Varano B., Giammarioli S., De Meo S., Silecchia G. (2016). Visceral fat adipocytes from obese and colorectal cancer subjects exhibit distinct secretory and ω6 polyunsaturated fatty acid profiles and deliver immunosuppressive signals to innate immunity cells. Oncotarget.

[6] Donninelli G., Del Cornò M., Pierdominici M., Scazzocchio B., Varì R., Varano B., Pacella I., Piconese S., Barnaba V., D’Archivio M. (2017). Distinct Blood and Visceral Adipose Tissue Regulatory T Cell and Innate Lymphocyte Profiles Characterize Obesity and Colorectal Cancer. Front. Immunol..

[7] Thanikachalam K., Khan G. (2019). Colorectal Cancer and Nutrition. Nutrients.

[8] Hull M.A. (2021). Nutritional prevention of colorectal cancer. Proc. Nutr. Soc..

[9] Mallick R., Basak S., Das R.K., Banerjee A., Paul S., Pathak S., Duttaroy A.K. (2023). Fatty Acids and Their Proteins in Adipose Tissue Inflammation. Cell Biochem. Biophys..

[10] Del Cornò M., Varì R., Scazzocchio B., Varano B., Masella R., Conti L. (2021). Dietary Fatty Acids at the Crossroad between Obesity and Colorectal Cancer: Fine Regulators of Adipose Tissue Homeostasis and Immune Response. Cells.

[11] Harwood J.L. (2023). Polyunsaturated Fatty Acids: Conversion to Lipid Mediators, Roles in Inflammatory Diseases and Dietary Sources. Int. J. Mol. Sci..

[12] Moon Y.A. (2023). Emerging roles of polyunsaturated fatty acid synthesis pathway in colorectal cancer. Anim. Cells Syst..

[13] Khan I., Hussain M., Jiang B., Zheng L., Pan Y., Hu J., Khan A., Ashraf A., Zou X. (2023). Omega-3 long-chain polyunsaturated fatty acids: Metabolism and health implications. Prog. Lipid Res..

[14] Calder P.C. (2017). Omega-3 fatty acids and inflammatory processes: From molecules to man. Biochem. Soc. Trans..

[15] Ulven S.M., Christensen J.J., Nygård O., Svardal A., Leder L., Ottestad I., Lysne V., Laupsa-Borge J., Ueland P.M., Midttun Ø. (2019). Using metabolic profiling and gene expression analyses to explore molecular effects of replacing saturated fat with polyunsaturated fat-a randomized controlled dietary intervention study. Am. J. Clin. Nutr..

[16] Bouwens M., van de Rest O., Dellschaft N., Bromhaar M.G., de Groot L.C., Geleijnse J.M., Müller M., Afman L.A. (2009). Fish-oil supplementation induces antiinflammatory gene expression profiles in human blood mononuclear cells. Am. J. Clin. Nutr..

[17] Bouwens M., Grootte Bromhaar M., Jansen J., Müller M., Afman L.A. (2010). Postprandial dietary lipid-specific effects on human peripheral blood mononuclear cell gene expression profiles. Am. J. Clin. Nutr..

[18] Skulas-Ray A.C., Kris-Etherton P.M., Harris W.S., Vanden Heuvel J.P., Wagner P.R., West S.G. (2011). Dose-response effects of omega-3 fatty acids on triglycerides, inflammation, and endothelial function in healthy persons with moderate hypertriglyceridemia. Am. J. Clin. Nutr..

[19] Myhrstad M.C., Ulven S.M., Günther C.C., Ottestad I., Holden M., Ryeng E., Borge G.I., Kohler A., Brønner K.W., Thoresen M. (2014). Fish oil supplementation induces expression of genes related to cell cycle, endoplasmic reticulum stress and apoptosis in peripheral blood mononuclear cells: A transcriptomic approach. J. Intern. Med..

[20] Rundblad A., Holven K.B., Bruheim I., Myhrstad M.C., Ulven S.M. (2018). Effects of fish and krill oil on gene expression in peripheral blood mononuclear cells and circulating markers of inflammation: A randomised controlled trial. J. Nutr. Sci..

[21] Rudkowska I., Ponton A., Jacques H., Lavigne C., Holub B.J., Marette A., Vohl M.C. (2011). Effects of a supplementation of n-3 polyunsaturated fatty acids with or without fish gelatin on gene expression in peripheral blood mononuclear cells in obese, insulin-resistant subjects. J. Nutr. Nutr..

[22] Rudkowska I., Paradis A.M., Thifault E., Julien P., Tchernof A., Couture P., Lemieux S., Barbier O., Vohl M.C. (2013). Transcriptomic and metabolomic signatures of an n-3 polyunsaturated fatty acids supplementation in a normolipidemic/normocholesterolemic Caucasian population. J. Nutr. Biochem..

[23] Polus A., Zapala B., Razny U., Gielicz A., Kiec-Wilk B., Malczewska-Malec M., Sanak M., Childs C.E., Calder P.C., Dembinska-Kiec A. (2016). Omega-3 fatty acid supplementation influences the whole blood transcriptome in women with obesity, associated with pro-resolving lipid mediator production. Biochim. Biophys. Acta.

[24] Tojjari A., Choucair K., Sadeghipour A., Saeed A. (2023). Anti-Inflammatory and Immune Properties of Polyunsaturated Fatty Acids (PUFAs) and Their Impact on Colorectal Cancer (CRC) Prevention and Treatment. Cancers.

[25] Itariu B.K., Zeyda M., Hochbrugger E.E., Neuhofer A., Prager G., Schindler K., Bohdjalian A., Mascher D., Vangala S., Schranz M. (2012). Long-chain n-3 PUFAs reduce adipose tissue and systemic inflammation in severely obese nondiabetic patients: A randomized controlled trial. Am. J. Clin. Nutr..

[26] Kratz M., Kuzma J.N., Hagman D.K., van Yserloo B., Matthys C.C., Callahan H.S., Weigle D.S. (2013). n3 PUFAs do not affect adipose tissue inflammation in overweight to moderately obese men and women. J. Nutr..

[27] Holt P.R., Alemán J.O., Walker J.M., Jiang C.S., Liang Y., de Rosa J.C., Giri D.D., Iyengar N.M., Milne G.L., Hudis C.A. (2017). Docosahexaenoic Acid Supplementation is Not Anti-Inflammatory in Adipose Tissue of Healthy Obese Postmenopausal Women. Int. J. Nutr..

[28] Huerta A.E., Prieto-Hontoria P.L., Fernández-Galilea M., Escoté X., Martínez J.A., Moreno-Aliaga M.J. (2017). Effects of dietary supplementation with EPA and/or α-lipoic acid on adipose tissue transcriptomic profile of healthy overweight/obese women following a hypocaloric diet. Biofactors.

[29] Ferguson J.F., Xue C., Hu Y., Li M., Reilly M.P. (2016). Adipose tissue RNASeq reveals novel gene-nutrient interactions following n-3 PUFA supplementation and evoked inflammation in humans. J. Nutr. Biochem..

[30] Shah R.D., Xue C., Zhang H., Tuteja S., Li M., Reilly M.P., Ferguson J.F. (2017). Expression of Calgranulin Genes S100A8, S100A9 and S100A12 Is Modulated by n-3 PUFA during Inflammation in Adipose Tissue and Mononuclear Cells. PLoS ONE.

[31] Fisk H.L., Childs C.E., Miles E.A., Ayres R., Noakes P.S., Paras-Chavez C., Kuda O., Kopecký J., Antoun E., Lillycrop K.A. (2022). Modification of subcutaneous white adipose tissue inflammation by omega-3 fatty acids is limited in human obesity-a double blind, randomised clinical trial. eBioMedicine.

[32] Murumalla R.K., Gunasekaran M.K., Padhan J.K., Bencharif K., Gence L., Festy F., Césari M., Roche R., Hoareau L. (2012). Fatty acids do not pay the toll: Effect of SFA and PUFA on human adipose tissue and mature adipocytes inflammation. Lipids Health Dis..

[33] Ferguson J.F., Roberts-Lee K., Borcea C., Smith H.M., Midgette Y., Shah R. (2019). Omega-3 polyunsaturated fatty acids attenuate inflammatory activation and alter differentiation in human adipocytes. J. Nutr. Biochem..

[34] Lee K.R., Midgette Y., Shah R. (2019). Fish Oil Derived Omega 3 Fatty Acids Suppress Adipose NLRP3 Inflammasome Signaling in Human Obesity. J. Endocr. Soc..

[35] Del Cornò M., Baldassarre A., Calura E., Conti L., Martini P., Romualdi C., Varì R., Scazzocchio B., D’Archivio M., Masotti A. (2019). Transcriptome Profiles of Human Visceral Adipocytes in Obesity and Colorectal Cancer Unravel the Effects of Body Mass Index and Polyunsaturated Fatty Acids on Genes and Biological Processes Related to Tumorigenesis. Front. Immunol..

[36] Maass P.G., Luft F.C., Bähring S. (2014). Long non-coding RNA in health and disease. J. Mol. Med..

[37] Shakhpazyan N.K., Mikhaleva L.M., Bedzhanyan A.L., Sadykhov N.K., Midiber K.Y., Konyukova A.K., Kontorschikov A.S., Maslenkina K.S., Orekhov A.N. (2023). Long Non-Coding RNAs in Colorectal Cancer: Navigating the Intersections of Immunity, Intercellular Communication, and Therapeutic Potential. Biomedicines.

[38] Lorente-Cebrián S., González-Muniesa P., Milagro F.I., Martínez J.A. (2019). MicroRNAs and other non-coding RNAs in adipose tissue and obesity: Emerging roles as biomarkers and therapeutic targets. Clin. Sci..

[39] Gao H., Kerr A., Jiao H., Hon C.C., Rydén M., Dahlman I., Arner P. (2018). Long Non-Coding RNAs Associated with Metabolic Traits in Human White Adipose Tissue. eBioMedicine.

[40] Sun L., Lin J.D. (2019). Function and Mechanism of Long Noncoding RNAs in Adipocyte Biology. Diabetes.

[41] Tait S., Baldassarre A., Masotti A., Calura E., Martini P., Varì R., Scazzocchio B., Gessani S., Del Cornò M. (2020). Integrated Transcriptome Analysis of Human Visceral Adipocytes Unravels Dysregulated microRNA-Long Non-coding RNA-mRNA Networks in Obesity and Colorectal Cancer. Front. Oncol..

[42] Djuricic I., Calder P.C. (2021). Beneficial Outcomes of Omega-6 and Omega-3 Polyunsaturated Fatty Acids on Human Health: An Update for 2021. Nutrients.

[43] Wang W.A., Groenendyk J., Michalak M. (2012). Calreticulin signaling in health and disease. Int. J. Biochem. Cell Biol..

[44] Mathers J.C. (2019). Obesity and bowel cancer: From molecular mechanisms to interventions. Nutr. Res..

[45] Tabuso M., Homer-Vanniasinkam S., Adya R., Arasaradnam R.P. (2017). Role of tissue microenvironment resident adipocytes in colon cancer. World J. Gastroenterol..

[46] Bernard J.J., Wellberg E.A. (2021). The Tumor Promotional Role of Adipocytes in the Breast Cancer Microenvironment and Macroenvironment. Am. J. Pathol..

[47] Holowatyj A.N., Haffa M., Lin T., Scherer D., Gigic B., Ose J., Warby C.A., Himbert C., Abbenhardt-Martin C., Achaintre D. (2020). Multi-Omics Analysis Reveals Adipose-tumor Crosstalk in Patients with Colorectal Cancer. Cancer Prev. Res..

[48] Alves M.G., Moreira Â., Guimarães M., Nora M., Sousa M., Oliveira P.F., Monteiro M.P. (2017). Body mass index is associated with region-dependent metabolic reprogramming of adipose tissue. BBA Clin..

[49] Fleckenstein-Elsen M., Dinnies D., Jelenik T., Roden M., Romacho T., Eckel J. (2016). Eicosapentaenoic acid and arachidonic acid differentially regulate adipogenesis, acquisition of a brite phenotype and mitochondrial function in primary human adipocytes. Mol. Nutr. Food Res..

[50] Engin A. (2017). Circadian Rhythms in Diet-Induced Obesity. Adv. Exp. Med. Biol..

[51] Kinouchi K., Sassone-Corsi P. (2020). Metabolic rivalry: Circadian homeostasis and tumorigenesis. Nat. Rev. Cancer.

[52] Ribas-Latre A., Eckel-Mahan K. (2022). Nutrients and the Circadian Clock: A Partnership Controlling Adipose Tissue Function and Health. Nutrients.

[53] Boden G., Duan X., Homko C., Molina E.J., Song W., Perez O., Cheung P., Merali S. (2008). Increase in endoplasmic reticulum stress-related proteins and genes in adipose tissue of obese, insulin-resistant individuals. Diabetes.

[54] Mak I.L., Lavery P., Agellon S., Rauch F., Murshed M., Weiler H.A. (2019). Arachidonic acid exacerbates diet-induced obesity and reduces bone mineral content without impacting bone strength in growing male rats. J. Nutr. Biochem..

[55] Xing C., Sun S.G., Yue Z.Q., Bai F. (2021). Role of lncRNA LUCAT1 in cancer. Biomed. Pharmacother..

[56] Wang Y., Hong Z., Wei S., Ye Z., Chen L., Qiu C. (2023). Investigating the role of LncRNA PSMG3-AS1 in gastric cancer: Implications for prognosis and therapeutic intervention. Cell Cycle.

[57] Luo D., Liu Y., Yuan S., Bi X., Yang Y., Zhu H., Li Z., Ji L., Yu X. (2022). The emerging role of NR2F1-AS1 in the tumorigenesis and progression of human cancer. Pathol. Res. Pract..

[58] Lu X., Yu Y., Liao F., Tan S. (2019). Homo Sapiens Circular RNA 0079993 (hsa_circ_0079993) of the POLR2J4 Gene Acts as an Oncogene in Colorectal Cancer Through the microRNA-203a-3p.1 and CREB1 Axis. Med. Sci. Monit..

[59] Lu C., Liao W., Huang Y., Luo Y. (2022). Increased expression of NOP14 is associated with improved prognosis due to immune regulation in colorectal cancer. BMC Gastroenterol..

[60] Ren H., Li Z., Tang Z., Li J., Lang X. (2020). Long noncoding MAGI2-AS3 promotes colorectal cancer progression through regulating miR-3163/TMEM106B axis. J. Cell. Physiol..

[61] Ma L., Gao J., Zhang N., Wang J., Xu T., Lei T., Zou X., Wei C., Wang Z. (2022). Long noncoding RNA SNHG17: A novel molecule in human cancers. Cancer Cell Int..

[62] Wu Y., Wang Z., Yu S., Liu D., Sun L. (2022). LncmiRHG-MIR100HG: A new budding star in cancer. Front. Oncol..

[63] Liu J.X., Li W., Li J.T., Liu F., Zhou L. (2018). Screening key long non-coding RNAs in early-stage colon adenocarcinoma by RNA-sequencing. Epigenomics.

[64] Peng J., Ma Y., Zhao X., Yang X., Wang H. (2022). Constitutive β-Catenin Overexpression Represses Lncrna MIR100HG Transcription via HDAC6-Mediated Histone Modification in Colorectal Cancer. Mol. Cancer Res..

[65] Thunen A., La Placa D., Zhang Z., Shively J.E. (2022). Role of lncRNA LIPE-AS1 in adipogenesis. Adipocyte.

[66] Zhu J., Jiang C., Hui H., Sun Y., Tao M., Liu Y., Qian X. (2022). Overexpressed lncRNA LINC00893 Suppresses Progression of Colon Cancer by Binding with miR-146b-3p to Upregulate PRSS8. J. Oncol..

[67] Pan S., Chen Y., Yan J., Li F., Chen X., Xu X., Xing H. (2022). The emerging roles and mechanisms of exosomal non-coding RNAs in the mutual regulation between adipose tissue and other related tissues in obesity and metabolic diseases. Front. Endocrinol..

[68] Norreen-Thorsen M., Struck E.C., Öling S., Zwahlen M., Von Feilitzen K., Odeberg J., Lindskog C., Pontén F., Uhlén M., Dusart P.J. (2022). A human adipose tissue cell-type transcriptome atlas. Cell Rep..

[69] Rey F., Urrata V., Gilardini L., Bertoli S., Calcaterra V., Zuccotti G.V., Cancello R., Carelli S. (2021). Role of long non-coding RNAs in adipogenesis: State of the art and implications in obesity and obesity-associated diseases. Obes. Rev..

[70] Squillaro T., Peluso G., Galderisi U., Di Bernardo G. (2020). Long non-coding RNAs in regulation of adipogenesis and adipose tissue function. eLife.

[71] Giuliani A., Ferrara F., Scimò M., Angelico F., Olivieri L., Basso L. (2014). Adipose tissue fatty acid composition and colon cancer: A case-control study. Eur. J. Nutr..

[72] Cottet V., Vaysse C., Scherrer M.L., Ortega-Deballon P., Lakkis Z., Delhorme J.B., Deguelte-Lardière S., Combe N., Bonithon-Kopp C. (2015). Fatty acid composition of adipose tissue and colorectal cancer: A case-control study. Am. J. Clin. Nutr..

[73] Vaittinen M., Männistö V., Käkelä P., Ågren J., Tiainen M., Schwab U., Pihlajamäki J. (2017). Interorgan cross talk between fatty acid metabolism, tissue inflammation, and FADS2 genotype in humans with obesity. Obesity.

[74] Garaulet M., Pérez-Llamas F., Pérez-Ayala M., Martínez P., de Medina F.S., Tebar F.J., Zamora S. (2001). Site-specific differences in the fatty acid composition of abdominal adipose tissue in an obese population from a Mediterranean area: Relation with dietary fatty acids, plasma lipid profile, serum insulin, and central obesity. Am. J. Clin. Nutr..

[75] Arner P., Rydén M. (2015). Fatty Acids, Obesity and Insulin Resistance. Obes Facts.

[76] Hodson L., McQuaid S.E., Karpe F., Frayn K.N., Fielding B.A. (2009). Differences in partitioning of meal fatty acids into blood lipid fractions: A comparison of linoleate, oleate, and palmitate. Am. J. Physiol. Endocrinol. Metab..

[77] Zhang L., Han L., He J., Lv J., Pan R., Lv T. (2020). A high serum-free fatty acid level is associated with cancer. J. Cancer Res. Clin. Oncol..

[78] Ameer F., Scandiuzzi L., Hasnain S., Kalbacher H., Zaidi N. (2014). De novo lipogenesis in health and disease. Metabolism.

[79] Worthmann A., Ridder J., Piel S.Y.L., Evangelakos I., Musfeldt M., Voß H., O’Farrell M., Fischer A.W., Adak S., Sundd M. (2024). Fatty acid synthesis suppresses dietary polyunsaturated fatty acid use. Nat. Commun..

[80] Roynette C.E., Calder P.C., Dupertuis Y.M., Pichard C. (2004). n-3 polyunsaturated fatty acids and colon cancer prevention. Clin. Nutr..

[81] González-Becerra K., Ramos-Lopez O., Barrón-Cabrera E., Riezu-Boj J.I., Milagro F.I., Martínez-López E., Martínez J.A. (2019). Fatty acids, epigenetic mechanisms and chronic diseases: A systematic review. Lipids Health Dis..

[82] Kim D., Langmead B., Salzberg S.L. (2015). HISAT: A fast spliced aligner with low memory requirements. Nat. Methods.

[83] Pertea M., Pertea G.M., Antonescu C.M., Chang T.C., Mendell J.T., Salzberg S.L. (2015). StringTie enables improved reconstruction of a transcriptome from RNA-seq reads. Nat. Biotechnol..

[84] Pertea G., Pertea M. (2020). GFF Utilities: GffRead and GffCompare. F1000Research.

[85] R Core Team (2021). R: A Language and Environment for Statistical Computing.

[86] Robinson M.D., McCarthy D.J., Smyth G.K. (2010). edgeR: A Bioconductor package for differential expression analysis of digital gene expression data. Bioinformatics.

[87] Risso D., Ngai J., Speed T.P., Dudoit S. (2014). Normalization of RNA-seq data using factor analysis of control genes or samples. Nat. Biotechnol..

[88] Durinck S., Spellman P.T., Birney E., Huber W. (2009). Mapping identifiers for the integration of genomic datasets with the R/Bioconductor package biomaRt. Nat. Protoc..

[89] Li J.H., Liu S., Zhou H., Qu L.H., Yang J.H. (2014). starBase v2.0: Decoding miRNA-ceRNA, miRNA-ncRNA and protein-RNA interaction networks from large-scale CLIP-Seq data. Nucleic Acids Res..

[90] Lin Y., Liu T., Cui T., Wang Z., Zhang Y., Tan P., Huang Y., Yu J., Wang D. (2020). RNAInter in 2020: RNA interactome repository with increased coverage and annotation. Nucleic Acids Res..

[91] Shannon P., Markiel A., Ozier O., Baliga N.S., Wang J.T., Ramage D., Amin N., Schwikowski B., Ideker T. (2003). Cytoscape: A software environment for integrated models of biomolecular interaction networks. Genome Res..

[92] Bindea G., Mlecnik B., Hackl H., Charoentong P., Tosolini M., Kirilovsky A., Fridman W.H., Pagès F., Trajanoski Z., Galon J. (2009). ClueGO: A Cytoscape plug-in to decipher functionally grouped gene ontology and pathway annotation networks. Bioinformatics.

