# Supplementary material for: Gene and lncRNA Profiling of ω3/ω6 Polyunsaturated Fatty Acid-Exposed Human Visceral Adipocytes Uncovers Different Responses in Healthy Lean, Obese and Colorectal Cancer-Affected Individuals
Source: Int J Mol Sci. 2024 Mar 15;25(6):3357. doi: 10.3390/ijms25063357 (PMC10970109; doi:10.3390/ijms25063357)
Supplement: Supplementary file 1 [file ijms-25-03357-s001.zip › Supplemental Data 1-3-4.pdf]

Supplemental Data S1. qPCR analysis of 6 randomly chosen genes affected in NW individuals by PUFA treatments**.** Results are expressed as the fold change (FC) with respect to the corresponding untreated control. p-values ≤ 0.05 are in bold; Cells colored in light red or blue indicate the concordance with up- or down-regulation in RNASeq data, respectively.

**Gene**	**NW-AA**		**NW-DHA**		**OB-AA**		**OB-DHA**		**CRC-AA**		**CRC-DHA**	
	FC	p-value	FC	p-value	FC	p-value	FC	p-value	FC	p-value	FC	p-value
**ACTB**	1.92	**0.042**	2.17	0.095	3.24	0.934	0.51	0.130	1.95	0.186	0.96	0.517
**CAST**	2.01	**0.002**	1.84	0.110	1.67	**0.007**	1.10	0.916	1.30	0.374	1.20	0.882
**DDR1**	1.42	0.206	1.56	**0.017**	0.60	0.333	0.55	**0.007**	1.79	0.397	1.38	0.725
**FAT1**	1.58	**0.001**	1.55	**0.019**	1.71	0.148	0.79	0.270	1.92	0.392	1.52	0.754
**LIPE**	0.39	**0.002**	0.50	**0.009**	2.30	0.977	1.13	0.627	0.56	**0.022**	0.39	**0.045**
**SLC2A5**	6.58	**0.005**	7.02	**0.0014**	1.85	0.82	0.74	0.331	9.04	0.066	6.08	0.058

Supplemental Data S3. Anthropometric characteristics of subjects included in the study**.**
Data are mean ±Standard Error. BMI, body mass index.

	**Lean**	**Obese**	**Cancer**
N (Male/Female)	3 (0M, 3F)	3 (2M, 1F)	3 (2M, 1F)
Age (years)	36,3±4,4	44,7±8,8	57,7±4,7
Body weight (Kg)	60,3±5,7	104,3±33,5	70±12,2
BMI (Kg/m^2)	23±1,4	35,2±5	23,3±1,4

Supplemental Data S4: List of genes and probes selected for real time qPCR analysis.

**Gene Symbol**	**Gene Name**	**Assay ID**
ACTB	actin b	Hs99999903_m1
CAST	calpastatin	Hs00156280_m1
DDR1	discoidin domain receptor tyrosine kinase 1	Hs01058430_m1
FAT1	FAT atypical cadherin 1	Hs00170627_m1
LIPE	lipase E, hormone sensitive type	Hs00943410_m1
SLC2A5	solute carrier family 2 member 5	Hs01086390_m1
